# To embolize or not to embolize: that is the question for arteriovenous malformations

**DOI:** 10.3171/2020.10.FOCVID20106

**Published:** 2021-01-01

**Authors:** Mustafa K. Baskaya, Angela M. Richardson

**Affiliations:** Department of Neurological Surgery, University of Wisconsin–Madison, Wisconsin

Dowlati et al. presented a case of a cerebellopontine angle, Spetzler-Martin grade II arteriovenous malformation (AVM), where they clearly demonstrated the key surgical points.^1^ We commend the authors on the clinical and radiologic outcome for this patient. The patient initially presented with AVM rupture and underwent external ventricular drain placement and suboccipital decompression with hematoma evacuation. Following a diagnostic angiogram to better characterize the AVM, he was treated with two stages of embolization, without complication, at an outside hospital. However, the embolic material did not completely penetrate the AVM nidus. Follow-up angiography at 4 months demonstrated continued arteriovenous shunting and recruitment of additional dural feeders. Resection was thus recommended and was completed via a retrosigmoid craniotomy. The patient recovered well, with postoperative angiography demonstrating complete resolution of the AVM.^1^ Fortunately, this patient recovered well. He had no complications from his diagnostic angiogram, from either the embolization procedure or the definitive microsurgical resection of the AVM. Diagnostic angiography is a key step in the workup for cerebral AVMs to define the feeding vessels and venous drainage and identify any high-risk features. Controversy remains regarding the role of embolization in the treatment of cerebral AVMs. All procedures—endovascular or surgical—carry risk. The question with AVMs is, is the risk of complications associated with embolization worth the benefit?

Embolization of cerebral AVMs may be performed for several reasons. AVMs may be embolized with curative intent, as a preoperative adjunct, as an adjunctive treatment prior to radiosurgery, or for palliative treatment. The choice to embolize, and for what purpose, is guided by the nature of each particular AVM, where the feeders are, and the Spetzler-Martin grade. In one series of AVM embolizations that were performed for any indication, the rate of mortality and permanent neurologic morbidity was 7.5% per patient.^2^ Another series, which examined the use of Onyx for embolization, evaluated 82 consecutive patients. In this cohort, disabling morbidity occurred in 3.8% of the patients and mortality due to hemorrhage in 2.4%, with an overall 11.3% rate of permanent morbidity and mortality related to embolization procedures.^3^ A recent meta-analysis that included 8009 patients in 98 published papers found a periprocedural hemorrhage rate of 2.6% per procedure and 4.8% per patient. The morbidity associated with periprocedural hemorrhage was 14.6%, and the rate of mortality was 45%.^4^

Haw et al.^2^ reported on complications for embolizations performed for all indications, noting that complication profiles may differ by indication. The patient discussed in the Dowlati case, the subject of this editorial comment, underwent embolization with intent to cure. To examine the success rate of curative embolization, a systematic review by Wu et al.^5^ identified 15 studies that met inclusion criteria, consisting of 597 patients. Of all the AVMs treated, 45.8% achieved complete obliteration, with stable occlusion reported in 96% of those patients at 6 months. The overall clinical complication rate was 24.1%, with 1.5% procedure-related mortality.^5^ Given these data, the likelihood of cure with embolization does not appear to outweigh the risk from these procedures for the majority of AVMs. A role for curative embolization may only be justified for simple AVMs in deep inaccessible regions of the brain with endovascularly accessible feeding arteries, where surgery would carry excessive risk due to either eloquence or patient medical status.^6^

Embolization of AVMs may also be performed as a preoperative adjunct. Embolization to reduce blood flow to an AVM has been hypothesized to decrease blood loss and simplify surgery by eliminating arterial feeders. To examine this question, these authors analyzed 319 AVMs resected by a single neurosurgeon. Of these, 151 were treated with preoperative embolization. No difference was found in the blood loss during surgery for patients treated with embolization followed by surgery and patients treated with surgery alone. Additionally, resection times were longer for AVMs embolized preoperatively than for those treated with surgery alone. The interpretation of these data is complicated by the fact that AVMs that were embolized preoperatively were larger and more likely to have both superficial and deep venous drainage than those treated by surgery alone.^7^

In the past, the senior author (M.K.B.) used preoperative embolization in some selected cases to obliterate deep arterial feeders that are inaccessible during early exposure (i.e., lenticulostriates and thalamoperforators) or to stage reduction of arteriovenous shunting in very large high-flow AVMs for restoration of cerebral blood flow. Embolization was also used to reduce blood flow through the AVM, to make surgery safer for AVMs in or adjacent to eloquent areas.

Ongoing evaluation of the utility of preoperative embolization through accumulated experience has resulted in modifications of these indications and decreased the utilization of preoperative embolization. While preoperative embolization can treat a proximal ruptured aneurysm, the aneurysm can also be clipped during the same surgery that is used to resect the AVM, or later if necessary. Embolization is not required to treat deep feeders. These feeders can be ligated and divided during the course of the resection. Staging to decrease flow is not necessary for Spetzler-Martin grade I and II AVMs, nor in most grade III AVMs. Staging may have some use in higher-grade AVMs that require resection. In terms of making surgery easier, safer, or faster, neither the data above nor the senior author’s experience supports this hypothesis. While embolization may decrease bleeding near or in eloquent areas, using this adjunct does not meaningfully alter the surgical strategy or outcome, since dissection around the AVM is required in that area regardless.

As we have accumulated more experience operating on AVMs that were embolized preoperatively, we have made some observations that run counter to expectations. Embolization often leads to a good angiographic result with decreased flow through the AVM. However, in our experience, although feeders may appear completely obliterated on angiogram and may be visibly filled with Onyx at surgery, upon division of these vessels at surgery, they can still bleed. Additionally, one tenet of AVM surgery is to retract on the AVM and not on the surrounding brain. Dispersion of embolic material through the AVM may lead to decreased blood flow; however, this material then makes the nidus firmer, with increased difficulty of manipulation and visualization during the later stages of resection. This is particularly true in cases with a venous varix, which can thrombose after embolization, resulting in significant mass effect. Additionally, the risk of swelling following embolization but prior to planned microsurgical resection may necessitate surgery being performed emergently.

Embolization of AVMs has also been used prior to radiosurgery. Early data suggested that embolization prior to radiosurgery was a negative predictor of obliteration.^8^ A recent retrospective propensity-matched cohort analysis comparing 101 patients treated with embolization + stereotactic radiosurgery (SRS) or SRS alone refuted these findings, with similar rates of obliteration at 3, 4, 5, and 6 years posttreatment. However, the authors note symptomatic embolization-related complication rates of 8.3%.^9^ Without improvements in clinical outcomes, it remains difficult to justify an increased complication burden.

Palliative treatment of inoperable AVMs with partial embolization has also been suggested as an indication for embolization. One study compared patients receiving medical treatment with patients undergoing partial embolization and found that hemorrhage rates (25% and 45%, respectively) and worsening clinical status (31% and 27.3%, respectively) were similar between the groups.^10^

Based on our clinical experience operating on treatment-naïve and previously embolized AVMs, we believe that embolization is often not necessary. Embolization with curative intent is often not successful and carries significant risk. Palliative partial embolization of inoperable AVMs does not appear to provide protection from hemorrhage or clinical worsening. When embolization is used as an adjunct to radiosurgery or microsurgery, the combination of two procedures exposes the patient to cumulative risk and increases the cost of care. In order to recommend that a patient undergo embolization prior to definitive treatment, the cumulative risk of embolization plus surgery would need to be equal to or less than the risk of the surgery alone. Thus far, the data do not bear this out for the large majority of AVMs.

In our experience, embolization is not needed in Spetzler-Martin grade I, II, or III AVMs. Preoperative embolization may have a role in selected grade IV or V AVMs. However, this should not be an algorithm-based decision made solely on the basis of Spetzler-Martin grade. The decision to recommend preoperative embolization should be guided by surgical considerations based on the angioarchitecture, surgical approach, accessibility of specific feeders, AVM location, and the patient’s clinical status. This adjunct may be useful in carefully selected patients after close evaluation of preoperative angiograms by an experienced vascular neurosurgeon.
